# Supplementary material for: Resolving the full spectrum of human genome variation using Linked-Reads
Source: Genome Res. 2019 Apr;29(4):635–45. doi: 10.1101/gr.234443.118 (PMC6442396; doi:10.1101/gr.234443.118)
Supplement: Supplemental Material [file supp_gr.234443.118_Supplemental_longranger-2.2.zip › longranger-2.2.tar.bz2!/lr2.2-public/lib/stan/cmdstan/stan/doc/api/html/structstan_1_1gm_1_1statement.html]

Stan: stan::gm::statement Struct Reference


|  |  |
| --- | --- |
|  | Stan  2.5.0  probability, sampling & optimization |


- Main Page
- Related Pages
- Namespaces
- Classes
- Files

- Class List
- Class Index
- Class Hierarchy
- Class Members

All Classes Namespaces Files Functions Variables Typedefs Enumerations Enumerator Friends Macros Pages

- stan
- gm
- statement

Public Types |
Public Member Functions |
Public Attributes |
List of all members

stan::gm::statement Struct Reference

`#include <ast.hpp>`

|  |  |
| --- | --- |
| Public Types | |
| typedef boost::variant  < boost::recursive\_wrapper  < nil >  , boost::recursive\_wrapper  < assignment >  , boost::recursive\_wrapper  < sample >  , boost::recursive\_wrapper  < increment\_log\_prob\_statement >  , boost::recursive\_wrapper  < expression >  , boost::recursive\_wrapper  < statements >  , boost::recursive\_wrapper  < for\_statement >  , boost::recursive\_wrapper  < conditional\_statement >  , boost::recursive\_wrapper  < while\_statement >  , boost::recursive\_wrapper  < print\_statement >  , boost::recursive\_wrapper  < reject\_statement >  , boost::recursive\_wrapper  < return\_statement >  , boost::recursive\_wrapper  < no\_op\_statement > > | statement\_t |
|  | |

|  |  |
| --- | --- |
| Public Member Functions | |
|  | statement () |
|  | |
|  | statement (const statement\_t &st) |
|  | |
|  | statement (const nil &st) |
|  | |
|  | statement (const assignment &st) |
|  | |
|  | statement (const sample &st) |
|  | |
|  | statement (const increment\_log\_prob\_statement &st) |
|  | |
|  | statement (const expression &st) |
|  | |
|  | statement (const statements &st) |
|  | |
|  | statement (const for\_statement &st) |
|  | |
|  | statement (const conditional\_statement &st) |
|  | |
|  | statement (const while\_statement &st) |
|  | |
|  | statement (const print\_statement &st) |
|  | |
|  | statement (const reject\_statement &st) |
|  | |
|  | statement (const no\_op\_statement &st) |
|  | |
|  | statement (const return\_statement &st) |
|  | |
| bool | is\_no\_op\_statement () const |
|  | |

|  |  |
| --- | --- |
| Public Attributes | |
| statement\_t | statement\_ |
|  | |

## Detailed Description

Definition at line 634 of file ast.hpp.

## Member Typedef Documentation

typedef boost::variant<boost::recursive\_wrapper<nil>, boost::recursive\_wrapper<assignment>, boost::recursive\_wrapper<sample>, boost::recursive\_wrapper<increment\_log\_prob\_statement>, boost::recursive\_wrapper<expression>, boost::recursive\_wrapper<statements>, boost::recursive\_wrapper<for\_statement>, boost::recursive\_wrapper<conditional\_statement>, boost::recursive\_wrapper<while\_statement>, boost::recursive\_wrapper<print\_statement>, boost::recursive\_wrapper<reject\_statement>, boost::recursive\_wrapper<return\_statement>, boost::recursive\_wrapper<no\_op\_statement> > stan::gm::statement::statement\_t

Definition at line 648 of file ast.hpp.

## Constructor & Destructor Documentation

|  |  |  |  |  |
| --- | --- | --- | --- | --- |
| stan::gm::statement::statement | ( |  | ) |  |

Definition at line 1228 of file ast\_def.cpp.

|  |  |  |  |  |  |
| --- | --- | --- | --- | --- | --- |
| stan::gm::statement::statement | ( | const statement\_t & | *st* | ) |  |

Definition at line 1230 of file ast\_def.cpp.

|  |  |  |  |  |  |
| --- | --- | --- | --- | --- | --- |
| stan::gm::statement::statement | ( | const nil & | *st* | ) |  |

Definition at line 1231 of file ast\_def.cpp.

|  |  |  |  |  |  |
| --- | --- | --- | --- | --- | --- |
| stan::gm::statement::statement | ( | const assignment & | *st* | ) |  |

Definition at line 1232 of file ast\_def.cpp.

|  |  |  |  |  |  |
| --- | --- | --- | --- | --- | --- |
| stan::gm::statement::statement | ( | const sample & | *st* | ) |  |

Definition at line 1233 of file ast\_def.cpp.

|  |  |  |  |  |  |
| --- | --- | --- | --- | --- | --- |
| stan::gm::statement::statement | ( | const increment\_log\_prob\_statement & | *st* | ) |  |

Definition at line 1234 of file ast\_def.cpp.

|  |  |  |  |  |  |
| --- | --- | --- | --- | --- | --- |
| stan::gm::statement::statement | ( | const expression & | *st* | ) |  |

Definition at line 1236 of file ast\_def.cpp.

|  |  |  |  |  |  |
| --- | --- | --- | --- | --- | --- |
| stan::gm::statement::statement | ( | const statements & | *st* | ) |  |

Definition at line 1235 of file ast\_def.cpp.

|  |  |  |  |  |  |
| --- | --- | --- | --- | --- | --- |
| stan::gm::statement::statement | ( | const for\_statement & | *st* | ) |  |

Definition at line 1237 of file ast\_def.cpp.

|  |  |  |  |  |  |
| --- | --- | --- | --- | --- | --- |
| stan::gm::statement::statement | ( | const conditional\_statement & | *st* | ) |  |

Definition at line 1239 of file ast\_def.cpp.

|  |  |  |  |  |  |
| --- | --- | --- | --- | --- | --- |
| stan::gm::statement::statement | ( | const while\_statement & | *st* | ) |  |

Definition at line 1238 of file ast\_def.cpp.

|  |  |  |  |  |  |
| --- | --- | --- | --- | --- | --- |
| stan::gm::statement::statement | ( | const print\_statement & | *st* | ) |  |

Definition at line 1240 of file ast\_def.cpp.

|  |  |  |  |  |  |
| --- | --- | --- | --- | --- | --- |
| stan::gm::statement::statement | ( | const reject\_statement & | *st* | ) |  |

Definition at line 1241 of file ast\_def.cpp.

|  |  |  |  |  |  |
| --- | --- | --- | --- | --- | --- |
| stan::gm::statement::statement | ( | const no\_op\_statement & | *st* | ) |  |

Definition at line 1243 of file ast\_def.cpp.

|  |  |  |  |  |  |
| --- | --- | --- | --- | --- | --- |
| stan::gm::statement::statement | ( | const return\_statement & | *st* | ) |  |

Definition at line 1242 of file ast\_def.cpp.

## Member Function Documentation

|  |  |  |  |  |
| --- | --- | --- | --- | --- |
| bool stan::gm::statement::is\_no\_op\_statement | ( |  | ) | const |

Definition at line 1286 of file ast\_def.cpp.

## Member Data Documentation

statement\_t stan::gm::statement::statement\_

Definition at line 650 of file ast.hpp.

---

The documentation for this struct was generated from the following files:

- src/stan/gm/ast.hpp
- src/stan/gm/ast\_def.cpp


---

[ Stan Home Page ]

*© 2011–2014,
Stan Development Team.*
